# Respiratory virus coinfections during the COVID-19 pandemic: epidemiologic analysis and clinical outcomes from the Phase 2/3 molnupiravir trial (MOVe-OUT)

**DOI:** 10.1128/spectrum.03563-23

**Published:** 2024-02-01

**Authors:** Matthew G. Johnson, Julie M. Strizki, Erin Jensen, Jonathan Cohen, Christine Katlama, Roman Fishchuk, Alfredo Ponce-de-León, Nyda Fourie, Chien-Yu Cheng, Dorothy McCoy, Mary Vesnesky, Carmelle T. Norice, Ying Zhang, Angela Williams-Diaz, Michelle L. Brown, Patricia Carmelitano, Jay A. Grobler, Amanda Paschke, Carisa De Anda

**Affiliations:** 1 Merck & Co., Inc., Rahway, New Jersey, USA; 2 Jadestone Clinical Research, LLC, Rockville, Maryland, USA; 3 AP-HP. Sorbonne Université, Hôpital—Pitié Salpêtrière, Paris, France; 4 Université Toulouse III Paul Sabatier, CHU de Toulouse, Toulouse, France; 5 CNE Central City Clinical Hospital of Ivano-Frankivsk City Council, Ivano-Frankivsk, Ukraine; 6 Instituto Nacional de Ciencias Medicas y Nutrición Salvador Zubiran, Mexico City, Mexico; 7 IATROS International, Bloemfontein, South Africa; 8 Taoyuan General Hospital, Taoyuan, Taiwan; Wright State University, Dayton, Ohio, USA

**Keywords:** COVID-19, molnupiravir, epidemiology, respiratory pathogens

## Abstract

This exploratory *post hoc* analysis assessed the incidence of respiratory viral coinfections and their impact on clinical outcomes in non-hospitalized adults with mild-to-moderate coronavirus disease-2019 (COVID-19) treated with molnupiravir versus placebo for 5 days in the Phase 2/3 MOVe-OUT trial (NCT04575597), which took place in October 2020 to January 2021 (Phase 2, *n* = 302) and May 2021 to October 2021 (Phase 3, *n* = 1,433). Among 1,735 total randomized participants, 1,674 had a baseline respiratory pathogen panel (NxTAG Respiratory Pathogen Panel for the Luminex MAGPIX instrument) performed and 69 (4.1%) were coinfected with at least one additional respiratory viral pathogen. Human rhinovirus/enterovirus (39/69, 56.5%) was the most common coinfection detected at baseline. In the modified intention-to-treat population, two participants with coinfecting respiratory RNA viruses were hospitalized and received respiratory interventions through Day 29, and none died; one participant in the molnupiravir group was coinfected with human rhinovirus/enterovirus, and one participant in the placebo group was coinfected with human metapneumovirus. Hospitalization or death occurred in 6.2% and 9.0% of non-coinfected participants in the molnupiravir versus placebo group, respectively, and over 90% did not require respiratory interventions. Most coinfecting respiratory RNA viruses detected at baseline were not detected at the end of therapy in both the molnupiravir and placebo groups. In summary, participants coinfected with severe acute respiratory syndrome coronavirus 2 (SARS-CoV-2) and another respiratory RNA virus were not more likely to be hospitalized or die, or require respiratory interventions, compared to participants who were not coinfected with another respiratory RNA virus at baseline in both groups.

**IMPORTANCE:**

Respiratory viral coinfections are known to occur with coronavirus disease-2019 (COVID-19). In a cohort of non-hospitalized adults with mild-to-moderate COVID-19 treated with molnupiravir versus placebo in the MOVe-OUT trial during October 2020 to October 2021, 4.1% of participants had a documented viral coinfection; human rhinovirus/enterovirus was the most common pathogen detected with the NxTAG Respiratory Pathogen Panel assay. Participants who had a coinfection with severe acute respiratory syndrome coronavirus 2 (SARS-CoV-2) and another respiratory RNA virus were not more likely to have worse clinical outcomes compared to those participants without a viral coinfection, and many coinfecting respiratory RNA viruses were no longer detected at the end of the 5-day treatment period in both groups.

## INTRODUCTION

The reported incidence of respiratory virus coinfections with coronavirus disease-2019 (COVID-19) has varied from ~1% to 20%, depending on the pandemic phase, season, location, and patient population (1–9). There are complex interactions between respiratory viruses (e.g., enhanced or reduced replication or infectivity) and the host immune response (e.g., altered induction of inflammatory mediators) in coinfected patients, and the impact on clinical outcomes remains unclear (1–3, 7–11).

Molnupiravir is an oral nucleoside analog prodrug of β-D-N4-hydroxycytidine with activity against RNA viruses, including severe acute respiratory syndrome coronavirus 2 (SARS-CoV-2) (12–17). In MOVe-OUT (Phase 2: October 2020 to January 2021; Phase 3: May 2021 to October 2021; ClinicalTrials.gov NCT04575597), non-hospitalized adults ≥18 years old with mild-to-moderate COVID-19 were randomized to receive molnupiravir (Phase 2: 200, 400, or 800 mg; Phase 3: 800 mg) or placebo twice daily for 5 days (18, 19). The pivotal Phase 3 portion of MOVe-OUT demonstrated that a 5-day course of molnupiravir was safe and decreased the risk of hospitalization or death compared to placebo, with molnupiravir-treated participants also requiring fewer respiratory interventions (18–20). The objective of this exploratory *post hoc* analysis was to determine the incidence of respiratory viral coinfections in participants from MOVe-OUT and determine clinical outcomes in participants who were coinfected with respiratory RNA viruses.

## MATERIALS AND METHODS

A positive study-qualifying SARS-CoV-2 test was required and defined as a laboratory-confirmed test (PCR or other molecular test) performed locally with sample collection within 7 days (Phase 2) or 5 days (Phase 3) of randomization into the study. Nasopharyngeal swab specimens were collected at baseline (Day 1) and multiple post-baseline timepoints and sent to a central microbiology laboratory for confirmatory evaluation (18, 19). In addition to SARS-CoV-2 PCR testing, a respiratory pathogen panel (RPP, NxTAG Respiratory Pathogen Panel for the Luminex MAGPIX instrument, Quest Diagnostics Nichols Institute, Chantilly, VA) was used to identify potential respiratory pathogens at baseline and at Day 5 (end of therapy). Nucleic acids from the following pathogens could be detected and identified by the RPP assay: adenovirus; coronavirus 229E, OC43, NL63, or HKU1; human bocavirus (hBCV); human metapneumovirus (hMPV); influenza A, A H1, A H3, or B; human parainfluenza virus (hPIV) 1, 2, 3, or 4; respiratory syncytial virus (RSV) A or B; human rhinovirus/enterovirus (hRV/EV); *Chlamydophila pneumoniae*; and *Mycoplasma pneumoniae*. No additional testing for other respiratory pathogens was systematically performed for participants in the study.

Participants with a positive RPP result at baseline were considered coinfected in this analysis. All randomized participants were included in the epidemiologic analysis to comprehensively assess all potential respiratory coinfections based on the RPP assay. The incidence of coinfections, time course of enrollment and positive baseline RPPs per month, and detected respiratory pathogens were summarized. Clinical outcomes were assessed in the modified intention-to-treat population (MITT; all randomized participants who received at least one dose of study drug and were not hospitalized prior to the first dose), excluding participants who had an RPP positive for DNA viruses (e.g., hBCV) because molnupiravir targets RNA viruses. Participants with or without a positive RPP result for a respiratory RNA virus receiving molnupiravir or placebo were evaluated for hospitalization or death through Day 29, use of any respiratory interventions through Day 29, and repeat RPP testing at the end of therapy if they had a positive baseline RPP result.

## RESULTS

**Fig 1:**
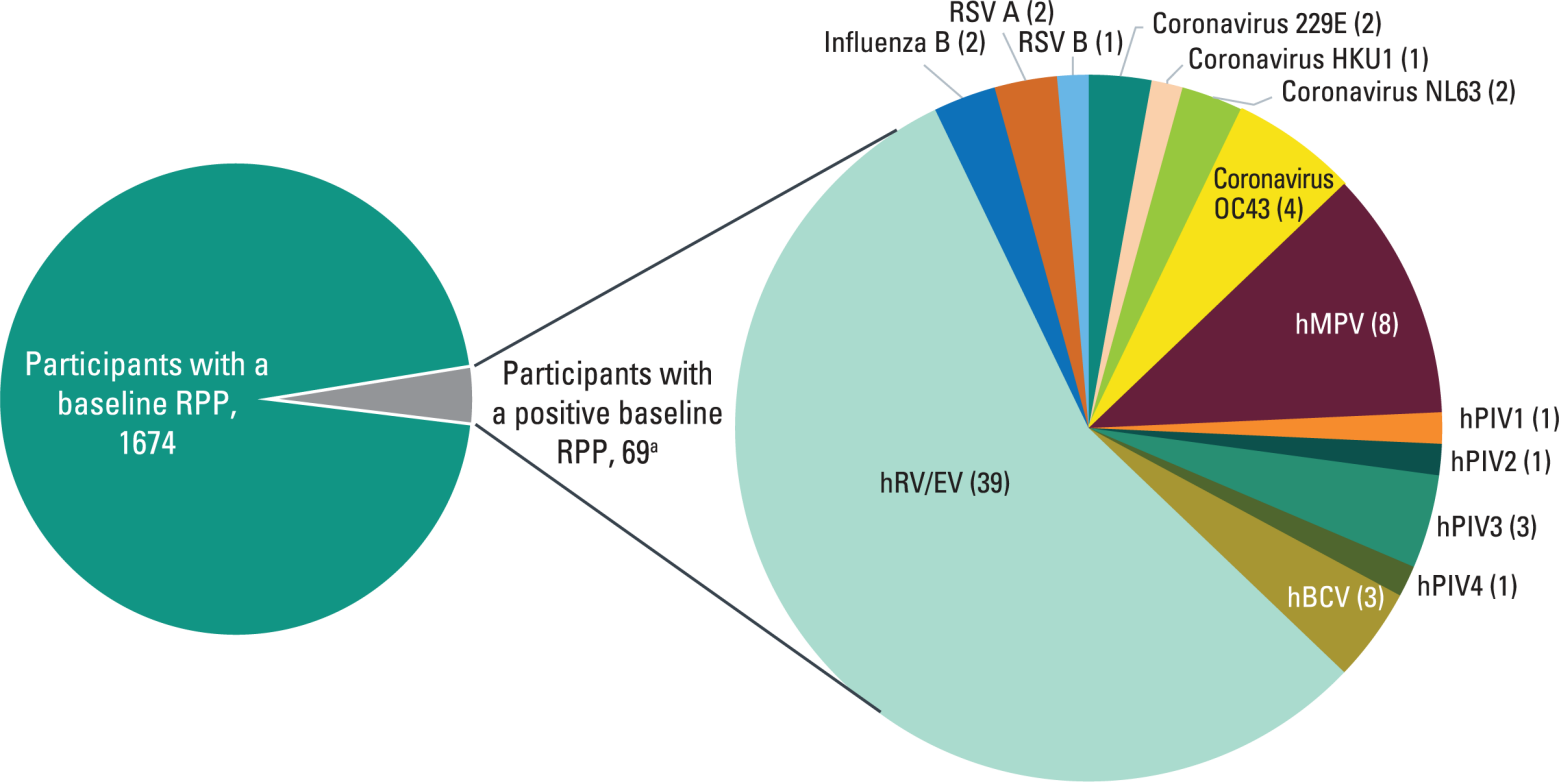
Number of non-SARS-CoV-2 respiratory pathogens detected at baseline (all randomized population). ^a^One participant had a baseline RPP positive for human rhinovirus/enterovirus and influenza B. Nucleic acids from the following pathogens could be detected and identified by the NxTAG Respiratory Pathogen Panel for the Luminex MAGPIX instrument: adenovirus; coronavirus 229E, OC43, NL63, or HKU1; human bocavirus; human metapneumovirus; influenza A, A H1, A H3, or B; human parainfluenza virus 1, 2, 3, or 4; respiratory syncytial virus A or B; human rhinovirus/enterovirus; *Chlamydophila pneumoniae*; and *Mycoplasma pneumoniae*.

Among the 1,735 participants randomized with mild-to-moderate COVID-19 in MOVe-OUT (Phase 2: *n* = 302; Phase 3: *n* = 1,433), 96.5% (1,674/1,735) had a baseline RPP performed and 4.1% (69/1,674) had at least one non-SARS-CoV-2 respiratory pathogen detected at baseline. The highest number of coinfections occurred in September 2021 (Table 1). hRV/EV (*n* = 39) was the most frequently detected respiratory pathogen, followed by non-SARS-CoV-2 coronaviruses (*n* = 9), hMPV (*n* = 8), and hPIV (*n* = 6) (Fig. 1).

**TABLE 1:** Monthly enrollment and monthly positive RPPs (all randomized population)^*a*^

Study phase	Month	No. of participants enrolled per month	No. of participants with a positive RPP per month
2	October 2020	6	0
	November 2020	79	4
	December 2020	172	5
	January 2021	45	2
3	May 2021	83	6
	June 2021	276	9
	July 2021	353	12
	August 2021	375	7
	September 2021	331	22
	October 2021	15	1

^*a*^ Phase 2: 19 October 2020 to 9 January 2021 and Phase 3: 6 May 2021 to 2 October 2021.

**Fig 2:**
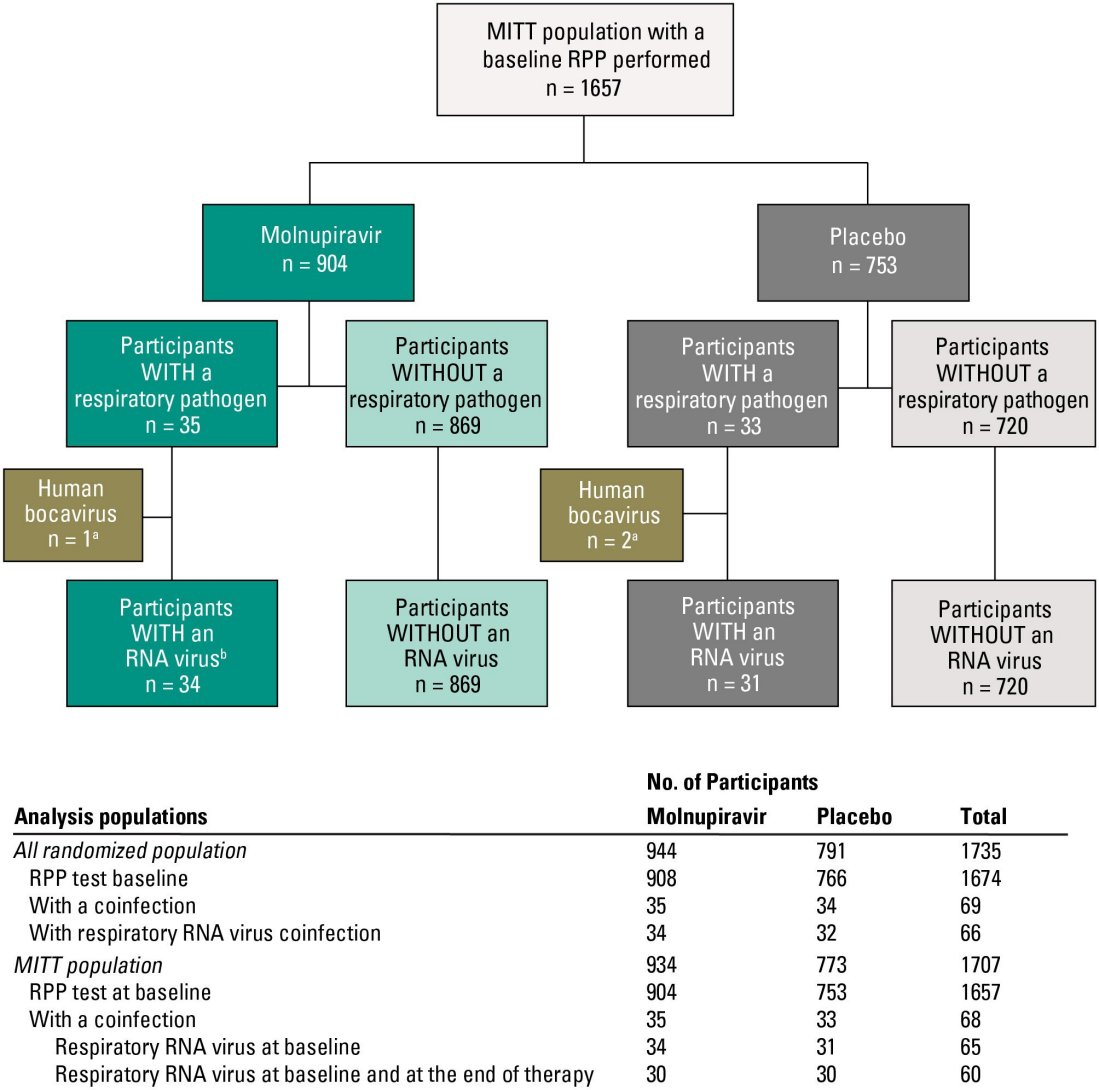
Flow of participants (MITT population). ^a^DNA viruses were excluded from this analysis. ^b^One participant had a baseline RPP positive for human rhinovirus/enterovirus and influenza B.

In the MITT population with a baseline RPP (1,657 participants), 65 participants (34 molnupiravir-treated and 31 placebo-treated) (Table 2) were coinfected with a non-SARS-CoV-2 respiratory RNA virus (Fig. 2). Baseline demographics and characteristics of participants with and without respiratory RNA virus coinfections with COVID-19 were generally comparable (Table 2); but compared with participants who were not coinfected, a higher proportion of coinfected participants were randomized ≥4 days after symptom onset [44/65 (67.7%) versus 882/1,589 (55.5%)], had moderate COVID-19 [36/65 (55.4%) versus 703/1,589 (44.2%)], and had undetectable SARS-CoV-2 RNA at baseline per the central microbiology laboratory [22/65 (33.8%) versus 123/1,589 (7.7%)] despite a positive study-qualifying SARS-CoV-2 test performed locally during screening. Of the 22 coinfected participants with undetectable SARS-CoV-2 RNA at baseline, the following coinfections were detected: hRV/EV (13, 59.1%), hPIV 3 (3, 13.6%), coronavirus OC43 (2, 9.1%), hMPV (1, 4.5%), influenza B (1, 4.5%), RSV A (1, 4.5%), and RSV B (1, 4.5%).

**TABLE 2:** Baseline demographics and characteristics of participants with and without respiratory RNA virus coinfections (MITT population)

Demographics and characteristics	Coinfected with a respiratory RNA virus		Not coinfected with a respiratory RNA virus	
	Molnupiravir (*n* = 34)	Placebo (*n* = 31)	Molnupiravir (*n* = 869)	Placebo (*n* = 720)
Sex, *n* (%)				
Male	12 (35.3)	15 (48.4)	416 (47.9)	367 (51.0)
Female	22 (64.7)	16 (51.6)	453 (52.1)	353 (49.0)
Age				
Median (range)	42 (25–82)	43 (21–83)	45 (18–90)	44 (18–88)
≤60 years, *n* (%)	29 (85.3)	27 (87.1)	705 (81.1)	587 (81.5)
Race, *n* (%)				
White	18 (52.9)	20 (64.5)	525 (60.4)	426 (59.2)
Multiple	11 (32.4)	8 (25.8)	208 (23.9)	190 (26.4)
American Indian or Alaska Native	4 (11.8)	1 (3.2)	60 (6.9)	46 (6.4)
Black or African American	0 (0.0)	2 (6.5)	51 (5.9)	35 (4.9)
Asian	1 (2.9)	0 (0.0)	25 (2.9)	22 (3.1)
Ethnicity				
Hispanic or Latino, *n* (%)	19 (55.9)	16 (51.6)	399 (45.9)	349 (48.5)
Region				
Latin America	14 (41.2)	14 (45.2)	353 (40.6)	313 (43.5)
Europe	10 (29.4)	11 (35.5)	304 (35.0)	237 (32.9)
North America	7 (20.6)	3 (9.7)	107 (12.3)	70 (9.7)
Africa	3 (8.8)	3 (9.7)	86 (9.9)	84 (11.7)
Asia Pacific	0 (0.0)	0 (0.0)	19 (2.2)	16 (2.2)
Time from symptom onset to randomization, *n* (%)				
0 to 3 days	9 (26.5)	12 (38.7)	377 (43.4)	330 (45.8)
4 to 5 days	20 (58.8)	19 (61.3)	424 (48.8)	366 (50.8)
>6 days	5 (14.7)	0 (0.0)	68 (7.8)	24 (3.3)
COVID-19 severity, *n* (%)				
Mild	15 (44.1)	14 (45.2)	484 (55.7)	402 (55.8)
Moderate	19 (55.9)	17 (54.8)	385 (44.3)	318 (44.2)
Risk factors for severe COVID-19, *n* (%)				
Body mass index ≥ 30	26 (76.5)	24 (77.4)	595 (68.5)	506 (70.3)
Age > 60 years	5 (14.7)	4 (12.9)	164 (18.9)	133 (18.5)
Diabetes mellitus	3 (8.8)	6 (19.4)	136 (15.7)	125 (17.4)
Serious heart condition	3 (8.8)	2 (6.5)	100 (11.5)	78 (10.8)
Chronic kidney disease	1 (2.9)	4 (12.9)	40 (4.6)	39 (5.4)
Chronic pulmonary disease	0 (0.0)	3 (9.7)	30 (3.5)	31 (4.3)
Active cancer	1 (2.9)	0 (0.0)	14 (1.6)	14 (1.9)
Baseline SARS-CoV-2 RNA NP^*a*^ sample, *n* (%)				
Detectable	24 (70.6)	17 (54.8)	766 (88.1)	652 (90.6)
Undetectable	10 (29.4)	12 (38.7)	75 (8.6)	48 (6.7)
Unknown	0 (0.0)	2 (6.5)	28 (3.2)	20 (2.8)
Baseline SARS-CoV-2 nucleocapsid antibody, *n* (%)				
Detectable	8 (23.5)	8 (25.8)	149 (17.1)	138 (19.2)
Undetectable	24 (70.6)	22 (71.0)	624 (71.8)	529 (73.5)
Unknown	2 (5.9)	1 (3.2)	96 (11.0)	53 (7.4)

^*a*^ NP, nasopharyngeal.

**Fig 3:**
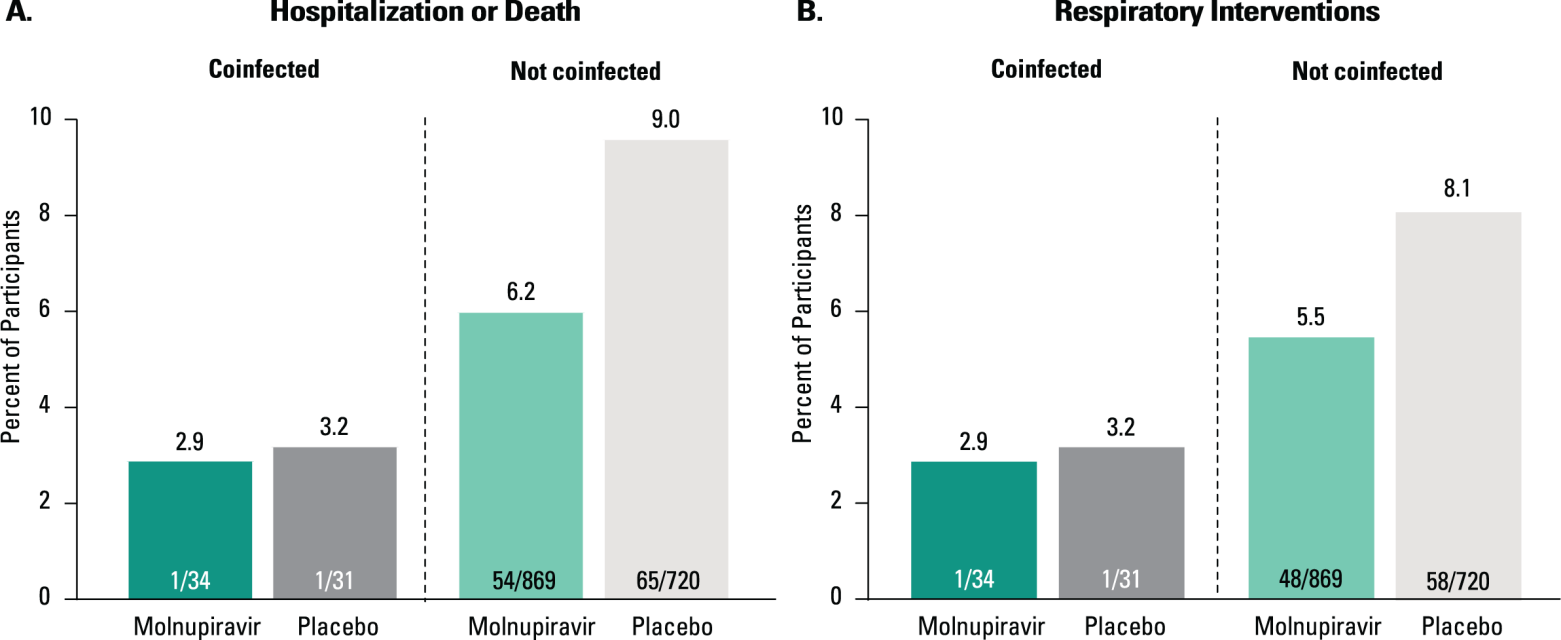
Hospitalization or death (**A**) and respiratory interventions (**B**) through Day 29 (MITT population). Unknown survival status at Day 29 was imputed as hospitalization or death for one participant in the placebo group who was not coinfected with a respiratory RNA virus at baseline. Each participant was counted only once by the highest level of oxygen therapy received. Respiratory interventions included conventional oxygen, high-flow heated and humidified device, non-invasive mechanical ventilation, and invasive mechanical ventilation.

Two participants (one in each treatment group) coinfected with respiratory RNA viruses, both of whom had detectable SARS-CoV-2 RNA at baseline per central laboratory testing, were hospitalized and received respiratory interventions. The participant hospitalized in the molnupiravir group (800 mg) was a 50-year-old female with moderate COVID-19 coinfected with hRV/EV who received oxygen via a high-flow heated and humidified device. The participant hospitalized in the placebo group was a 53-year-old female with mild COVID-19 coinfected with hMPV who received conventional oxygen therapy. These participants were alive at Day 29. Rates of hospitalization, death, and respiratory interventions were higher in both treatment groups among participants without a respiratory RNA virus coinfection. In participants who were not coinfected with a respiratory RNA virus, hospitalization occurred in 54 participants in the molnupiravir group, one of whom died. In the placebo group, 65 participants were hospitalized and 8 died. Ninety-five percent of non-coinfected participants in the molnupiravir group and 92% of participants in the placebo group did not require respiratory interventions (Fig. 3A and 3B).

**Fig 4:**
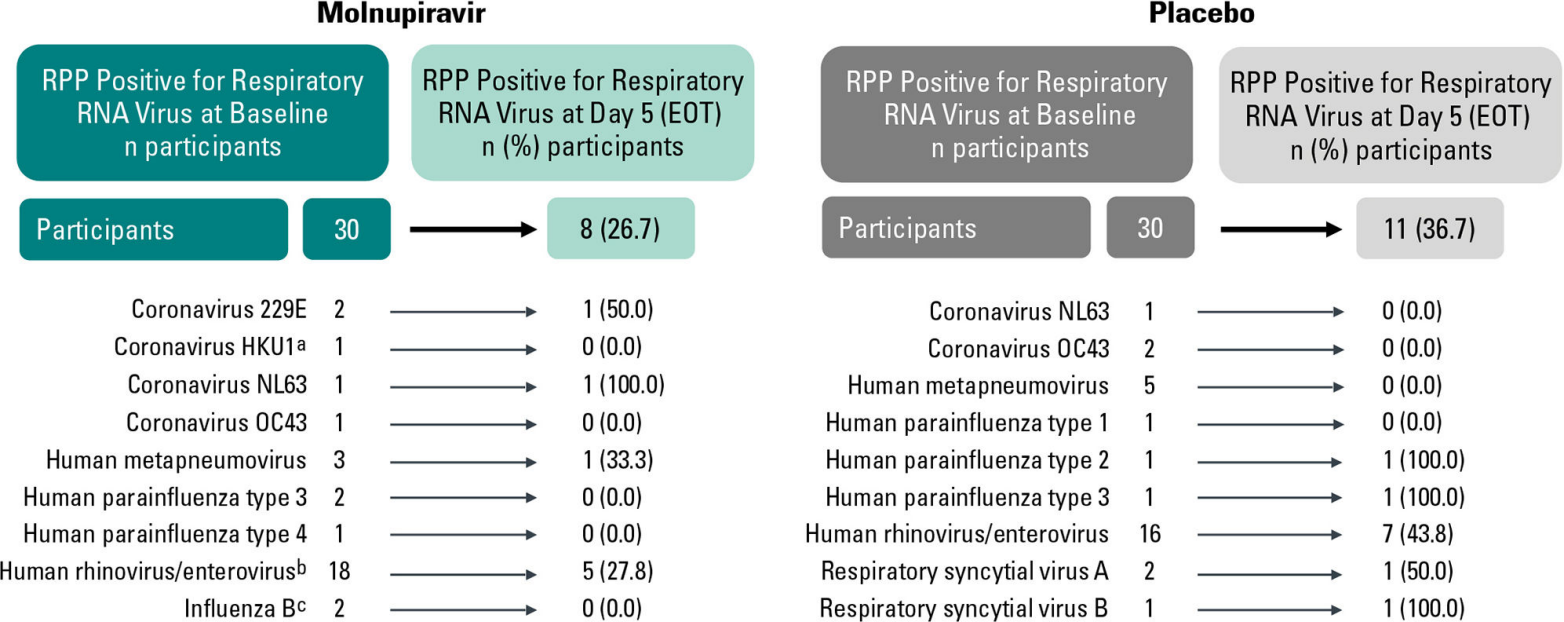
Respiratory RNA virus detection at the end of therapy (MITT population). Among the 65 participants in the MITT population with a respiratory RNA virus coinfection at baseline, 60 had a paired end of therapy RPP result. Participants received molnupiravir 800 mg twice daily unless otherwise noted. ^a^This participant received molnupiravir 400 mg twice daily. ^b^Six participants received molnupiravir 200 mg twice daily, and one participant received 400 mg twice daily. ^c^One participant received molnupiravir 200 mg twice daily. EOT, end of therapy.

Among participants with respiratory RNA virus coinfection at baseline and a paired RPP performed at the end of therapy in the MITT population (*n* = 60), 19 had non-SARS-CoV-2 viral RNA detected at the end of the 5-day treatment period (8 in the molnupiravir group and 11 in the placebo group) (Fig. 4).

## DISCUSSION

The results from this study add to the current literature on viral coinfections in patients with COVID-19. The incidence of respiratory viral coinfections in participants in MOVe-OUT (4.1%) and the frequent coinfection with hRV is comparable with other investigations (1–4, 6, 21). As identified in other epidemiologic analyses, coinfection with influenza and RSV were infrequent (two and three participants, respectively). Infection prevention interventions (e.g., social distancing, masking, quarantining) implemented worldwide which focused on interrupting virus transmission during the pandemic may have contributed to this finding (22, 23). Although the RPP in this study did not indicate the precise number of hRV coinfections (24, 25), since the assay only qualitatively reports the presence of hRV or hEV, it is possible that hRV coinfection contributed to the higher proportion of coinfected participants with undetectable SARS-CoV-2 RNA at baseline. hRV induces an interferon response that may reduce or prevent the replication of other respiratory viruses, including SARS-CoV-2, and impair their detection (26–29). This viral interference may have facilitated clearance of SARS-CoV-2 in the time between the positive study-qualifying and baseline SARS-CoV-2 test. Other factors such as timing of exposure, host immune status, and virus-virus interactions may have impacted coinfection rates with these other common respiratory pathogens (30).

We did not observe a higher proportion of hospitalization or death, or respiratory interventions, in coinfected versus non-coinfected participants, and most coinfecting respiratory RNA viruses detected at baseline were not detected at the end of therapy in both treatment groups. Molnupiravir has demonstrated *in vitro* and *in vivo* activity against non-SARS-CoV-2 RNA viruses, such as other coronaviruses (e.g., NL63, OC43, 229E), enteroviruses (e.g., A71), and influenza viruses (e.g., H1N1, H3N2, H5N1) (15, 31–35). However, the clinical efficacy of molnupiravir for the treatment of RNA viruses beyond SARS-CoV-2 has not been demonstrated to date.

This analysis was limited to the respiratory pathogens included in the RPP assay, which is not inclusive of all possible coinfecting viral, bacterial, or fungal respiratory pathogens. While some minor differences were noted in Table 2, such as a higher proportion of participants with moderate COVID-19 at baseline in the coinfected group compared to the non-coinfected group, the small number of participants coinfected with respiratory RNA viruses precludes definitive conclusions. Of note, some participants included in the molnupiravir group during the Phase 2 dose-ranging portion of the study received lower doses of molnupiravir (e.g., 200 mg and 400 mg) than the dose selected for the Phase 3 portion of the study (800 mg).

Given that certain coinfections such as SARS-CoV-2 and influenza (designated “flurona” due to seasonal cocirculation) have generated considerable scrutiny (36, 37), additional investigations are warranted to further elucidate the interactions between SARS-CoV-2 and other respiratory viruses, and the effects of molnupiravir on other RNA viruses. In closing, this subgroup analysis demonstrated that respiratory viral coinfections were infrequent during an earlier stage of the COVID-19 pandemic, coinfected participants were not more likely to have worse clinical outcomes compared to those participants without a viral coinfection, and the majority of viral coinfecting RNA viruses did not persist after the end of therapy.
